# Antimicrobial activity of *Hemidesmus indicus, Ficus bengalensis* and *Pterocarpus marsupium* roxb

**DOI:** 10.4103/0250-474X.58182

**Published:** 2009

**Authors:** M. Gayathri, K. Kannabiran

**Affiliations:** Biomolecules and Genetics Division, School of Biosciences and Technology, VIT University, Vellore-632 014, India

**Keywords:** Antimicrobial activity, *Ficus bengalensis*, *Hemidesmus indicus*, *Pterocarpus marsupium* roxb, saponins, tannins, saponins

## Abstract

The antimicrobial activity of *Hemidesmus indicus, Ficus bengalensis* and *Pterocarpus marsupium roxb* was evaluated against pathogenic bacteria *Stahylococcus aureus, Pseudomonas aeruginosa* and *Klebsiella pneumonia* in an *in vitro* condition. Aqueous extracts from roots of *H. indicus* and barks of *F. bengalensis* and *P. marspium roxb* were tested for antimicrobial activity using the zone of inhibition method and also screened for phytochemicals. The aqueous extract of *P. marsupium roxb* inhibited growth of bacteria with the minimal inhibitory concentration ranging from 0.04 mg to 0.08 mg and extracts of *F. bengalensis* and *H. indicus* showed inhibition at the range of 0.04 mg to 0.1 mg against the bacteria tested. The susceptibility of bacterial pathogens was in the order of *S. aureus, K. pneumoniae* and *P. aeruginosa.* The antimicrobial activity of plant extracts was synergistic with antibiotics tested. Results of the present study suggest that the aqueous extracts of *H. indicus, F. bengalensis* and *P. marspium* roxb has significant antibacterial activity against pathogenic bacteria.

Medicinal plants have been used as an exemplary source for centuries as an alternative remedy for treating human diseases because they contain numerous active constituents of therapeutic value[1]. The development of microbial resistance to antibiotics has led the researches to investigate the alternative sources for the treatment of resistant strains[2]. Presently 80 percent of the world population relies on plant derived medicines and serves as first line of defense in maintaining health and combating many diseases[3]. *H. indicus* serves as an alterative tonic, demulcent, diaphoretic and traditionally been used to treat venereal diseases, skin diseases, urinary infections, negative emotions and impotence[4]. It also prevents abdominal distention, arthritis, rheumatism, gout and epilepsy. *F. bengalensis* has long been used to treat vaginal complaints, fever, ulcers, erysipelas, vomiting, leprosy, inflammations, skin allergies, nose-diseases, gonorrhea, and inflammation of liver and serves as antidiarrheal, antidysenteric, hemostatic and antihemorrhoidal agent[5]. *P. marsupium roxb* has long been used as an antiinflammatory, antihelmenthic, antielephantiasis, antileucoderma agent and often used to treat dysentery, cough and diarrhea[6]. Hence, the aqueous extracts of roots of *H. indicus*, and the bark of *F. bengalensis* and *P. marsupium roxb* were chosen to study and their efficacy was tested with respect to inhibition over the growth of pathogenic bacteria under *in vitro* conditions.

The roots of *H. indicus,* bark of *F. bengalensis* and *P. marsupium roxb* were collected from the Morappur forest area, Dharmapuri District, Tamil Nadu during the month of April 2005. It was authenticated and the Voucher specimens were prepared and deposited in the Forest Department, Dharmapuri District, Tamil Nadu, India. Roots of *H. indicus* and stem barks of *F. bengalensis* and *P. marsupium roxb* were washed with distilled water, shade dried, powdered and stored in an air-tight container until for further use.

The powder (100 g) was used to prepare a juice in a Turmix electric extractor with 500 ml of distilled water. The juices were filtered and the residue was removed. The extracts were filter sterilized using syringe filter containing 0.22 μ cellulose acetate membrane filter (Sartorius) and concentrated under vacuum to get the solid and freeze dried.

A Gram positive bacteria *Staphylococcus aureus* (ATCC 700699) and two Gram negative bacteria *Pseudomonas aeruginosa* (ATCC 27853) and *Klebsiella pneumoniae* (ATCC 2719), were used as test organisms. Exactly 0.2 ml of overnight cultures of each organism was inoculated into 20 ml of sterile nutrient broth and incubated for 3-5 h and standardized to10^6^ cfu/ml. Mueller Hinton Agar solid media was used for culturing of bacteria. Agar diffusion assay was carried out to check the antimicrobial activity[7]. The plates were incubated at 37° for 24 h during which activity was evidenced by the presence of a zone of inhibition surrounding the well. Each test was repeated three times and the antibacterial activity was expressed as the mean of diameter of inhibition zones (mm) produced by the extracts when compared to antibiotics. The minimal inhibitory concentration (MIC) was carried out as described by serial dilution method[8]. Standard procedures were followed to identify the chemical constituents in the aqueous extract or the powdered specimens of the study plants[9].

*H. indicus* extracts (1 mg/ml) inhibited the growth of *S. aureus* and *K. pneumonia* (14 mm) and *P. aeruginosa* (12 mm). *F. bengalensis* and *P. marsupium roxb* were also showed a similar order of antimicrobial activity against the tested organisms (Table 1). The micro dilution analyses revealed that the *P. marsupium roxb* extract exhibited antibacterial activity with the MIC values ranging from 0.04 mg to 0.08 mg against tested organisms. *F. bengalensis* and *H. indicus* extracts exhibited moderate inhibition with the MIC ranging from 0.04 mg to 0.1 mg against tested bacterial pathogens (Table 2). Standard antibiotics ampicillin, tetracycline and chloromphenicol exhibited marked inhibition with the MIC values ranging from 0.013 and 0.03 mg/ml.

**TABLE 1:** ANTIMICROBIAL ACTIVITY OF THE PLANT EXTRACTS

Bacterial pathogens	Zone of Inhibition (mm)*		
	
	*H. indicus* (1 mg/ml)	*F. bengalensis* (1 mg/ml)	*P. marsupium* roxb (1 mg/ml)
*Staphylococcus aureus*	14±0.01	12±0.02	13±0.01
*Pseudomonas aeruginosa*	12±0.02	10±0.01	11±0.02
*Klebsiella pneumoniae*	14±0.01	13±0.01	12±0.01

* Values are mean±SD (n=3). Aqueous extract from the roots of *H. indicus* and from the bark of *F. bengalensis* and *P. marsupium* roxb was used for the assessment of antibacterial activity against selected bacterial pathogens.

**TABLE 2:** MINIMUM INHIBITORY CONCENTRATIONS OF THE AQUEOUS EXTRACTS

Bacterial pathogens	MIC (mg/ml)					
	
	*H. indicus*	*F. bengalensis*	*P. marsupium roxb*	AMP*	TTC+	COP¶
*Staphylococcus aureus*	0.1	0.1	0.08	0.03	0.03	0.03
*Pseudomonas aeruginosa*	0.04	0.04	0.04	0.013	0.013	0.013
*Klebsiella pneumoniae*	0.1	0.08	0.09	0.026	0.026	0.026

* AMP stands for ampicillin

+ TTC denotes tetracycline and

¶ COP is chloromphenicol. Aqueous extract of the roots of *H. indicus* and the bark of *F. bengalensis* and *P. marsupium* roxb was used to estimate the MIC values against selected bacterial pathogens.

Phytochemical screening of aqueous extracts revealed the presence of tannins, saponins, flavonoids, glycosides, phenolic compounds, carbohydrates and proteins. Quantitative analysis of phytochemicals in aqueous extracts of the roots of *H. indicus*, and the barks of *F. bengalensis* and *P. marsupium roxb* showed the presence of tannins (6.63, 7.75 and 6.15 mg/g, respectively) and saponins (3.01, 2.03 and 2.62 mg/g, respectively) as shown in Table 3. Tannins and saponins were the major phytochemicals present in the extracts.

**TABLE 3:** QUANTITATIVE ANALYSIS OF PHYTOCHEMICAL CONSTITUENTS

Phytochemicals	*H. indicus*	*F. bengalensis*	*P. marsupium* roxb
Total Phenol (mg/g)	0.15±0.32	0.22±0.19	0.13±0.17
Alkaloids (mg/g)	0.55±0.20	0.85±0.29	0.65±0.19
Tannins (mg/g)	6.63±0.19	7.75±0.20	6.15±0.25
Saponins (mg/g)	3.01±0.09	2.03±0.17	2.62±0.12
Flavonoids (mg/g)	0.98±0.09	0.57±0.32	0.63 ±0.12

Values are mean±SD of three determinations. Aqueous extract from the roots of *H. indicus* and from the bark of *F. bengalensis* and *P. marsupium* roxb was used for the estimation of phytochemicals.

The increase of antibiotic resistance by the pathogenic microorganisms to conventional drugs has necessitated the search for new, efficient and cost effective drugs for the control infectious diseases. Several reports have shown that the medicinal plants constitute a great source for the isolation of active drugs for the control of pathogenic organisms. The chloroform and ethanol (95%) extracts of roots of *H. indicus* have been shown to possess antifungal activity[10], further chloroform extract possess antibacterial effect against *H. pylori* from humans[11]. Antidiarrhoeal effect of methanol extract of *H. indicus* against *S. typhimurium, E. coli* and *S. flexneri* was already been reported in an experimentally-induced diarrhea in rats[12]. Antibacterial activity of chloroform and ethanol (95%) extracts of *H. indicus* roots was already been reported against different enterobacterial strains[13]. However, there is no report on the effect of aqueous root extract of *H. indicus* on pathogenic bacterial strains. The present study demonstrates that the *H. indicus* root extracts possess a significant antibacterial activity over selected pathogenic bacterial strains. The extent of inhibition of the bacterial growth was in the order of *H. indicus* >*P. marsupium* roxb>*F. bengalensis*. The MIC of plants extract required for the maximal antimicrobial activity was comparable with that of standard antibiotics tested.

Several reports are available in support of antibacterial activity of several phytochemicals present in plant extracts[14–16]. Antibacterial activity of tannins and saponnins isolated from plant species are well documented[17,18]. Larvicidal activity was reported for saponin isolated from fruit mesocarp of *Balanites aegyptiaca*[19]. However, further studies are needed to evaluate the antibacterial activity of isolated phytochemicals such as tannins and saponins from these plants against pathogenic bacterial strains.

In conclusion, aqueous extracts of the roots of *H. indicus* and the bark *F. bengalensis* and *P. marsupium Roxb* exhibited significant antibacterial activity against tested bacterial strains. Presence of tannins and saponins in higher concentration than the other phytochemicals suggests that these phytochemicals could likely be responsible for the antibacterial activity. However further studies are needed to establish that these plant extracts could form effective antimicrobial therapy against common bacterial diseases.
